# Occurrence of Comorbidities before and after Soft Tissue Sarcoma Diagnosis

**DOI:** 10.1155/2012/402109

**Published:** 2012-05-28

**Authors:** Myrthe P. P. van Herk-Sukel, Sumitra Shantakumar, Lucy I. H. Overbeek, Hester van Boven, Fernie J. A. Penning-van Beest, Ron M. C. Herings

**Affiliations:** ^1^PHARMO Institute for Drug Outcomes Research, P.O. Box 85222, 3508 AE Utrecht, The Netherlands; ^2^Oncology Biometrics and Epidemiology, Oncology R&D, GlaxoSmithKline, RTP, NC 27709, USA; ^3^The Nationwide Network and Registry for Histo- and Cytopathology in The Netherlands, Foundation PALGA, Utrecht, The Netherlands; ^4^The Netherlands Cancer Institute-Antoni van Leeuwenhoek Hospital (NKI-AVL), 1066 CX Amsterdam, The Netherlands; ^5^Department of Medical Informatics, Erasmus University Medical Centre, 3015 GE Rotterdam, The Netherlands

## Abstract

*Background*. Data is limited on the burden of common comorbidities, such as cardiovascular disease (CVD), respiratory disease and diabetes, or comorbidities related to cancer and its treatment, such as anemia and depression, in patients with soft tissue sarcoma (STS). *Patients and Methods*. From the Dutch Pathology Registry linked to the PHARMO database (including data on drug use and hospitalizations), 533 patients with STS were selected during 2000–2007 and matched 1 : 10 to cancer-free controls. The occurrences of comorbidities were assessed in the 12 months before and after STS diagnosis. *Results*. STS patients were 2–4 times more likely to have comorbidities at diagnosis compared with cancer-free controls. The incidence of CVD, anemia, and depression after STS diagnosis differed significantly from cancer-free controls and decreased during followup from 40–124 per 1,000 person-years (py) during the first six months to 11–38 per 1,000 py more than 12 months after diagnosis. The incidence of respiratory disease and diabetes among STS patients remained stable during followup (5–21 per 1,000 py) and did not differ significantly from cancer-free controls. *Conclusions*. STS patients were more likely to have comorbidities before cancer diagnosis and to develop CVD, anemia, and depression after diagnosis compared to cancer-free controls.

## 1. Introduction

Soft tissue sarcomas (STSs) are a rare and heterogeneous group of tumors arising from mesenchymal tissue [1]. They account for less than 1% of all new malignancies diagnosed in adults in Europe [2, 3]. STSs show a broad range of differentiation and may arise in, for example, fat (liposarcoma), muscle (smooth muscle: leiomyosarcoma and striated muscle: rhabdomyosarcoma), and fibrous tissue (e.g., fibrosarcoma, malignant fibrous hystiocytoma, and dermatofibrosarcoma) [1]. The most common treatment for STS is surgical resection with or without radiation therapy and chemotherapy is administered when metastases are present [4–6]. 

Little epidemiologic data on STS is available due to large variations in tumor biology, histology, location, stage at presentation, and the relative rarity of these tumors [7]. The development of STS has been shown to be associated with environmental factors, immunodeficiency, genetic factors, and viral infection [8, 9]. Important prognostic factors of STS reported in the literature include tumor size, histology, primary anatomic site, grade, and the presence of metastatic disease [8, 10, 11]. The overall survival of patients with STS has been improving, however this differs per histological subtype [12, 13]. Many of these patients will have concomitant conditions, which has various clinical implications. Comorbidities are independent determinants of prognosis (regardless of the chosen therapy) and their presence is also an indicator of the complexity of the clinical needs of each patient [14].

Previous studies using administrative healthcare databases showed that cardiovascular disease (CVD), respiratory disease, and diabetes are the most common concomitant illnesses in cancer patients [14, 15]. Currently, there is limited data available that describes the burden of these comorbidities among STS patients. It is important to quantify these comorbidities as these often necessitate modifications of and/or adversely influence the chosen therapy. Moreover, less is known about the impact of the most prevalent cancer symptom: fatigue [16]. Fatigue severely impacts quality of life and functional capacity. The condition itself is difficult to measure, but instead one can study treatable contributing factors like anemia and depression.

This retrospective cohort study describes the prevalence and incidence of comorbidities including cardiovascular disease, respiratory disease, diabetes, anemia, and depression among a population-based cohort of STS patients. These rates were compared to those observed in a matched noncancer control population. In addition, risk factors for developing these morbidities after STS diagnosis were determined.

## 2. Methods

### 2.1. Data Sources

Data from patients with a STS pathology specimen were obtained from PALGA, the Dutch Nationwide Network and Registry of Histo- and Cytopathology. PALGA contains data of all histological, cytological, and autopsy examinations in The Netherlands [17]. Currently, it contains about 42 million abstracts of all pathology reports with encrypted patient identification and diagnostic terms which are in scope with SNOMED classification. PALGA has achieved complete national coverage since 1990 and is the basis for the Dutch Cancer Registry. Records from PALGA were linked to the PHARMO record linkage system (PHARMO RLS). This linkage has been used in previous published studies [18, 19]. Data from the PHARMO RLS consist of multiple observational databases linked on a patient level, covering 3 million inhabitants of geographic defined areas in the Netherlands. Databases relevant for this study include the Dutch National Medical Register [20] and the community (out-patient) pharmacy database. The hospital records contain detailed information concerning admissions for more than 24 hours and admissions for less than 24 hours for which a bed is required, including primary and secondary diagnoses, procedures, and dates of hospital admission and discharge. All diagnoses are coded according to the International Classification of Diseases, Ninth Revision, Clinical Modification (ICD-9-CM). The drug dispensing histories from community pharmacies contain data on the dispensed drug, prescriber, dispensing date, amount dispensed, prescribed dose regimens, and thus the duration of use. All drugs are coded according to the Anatomical Therapeutic Chemical (ATC) Classification.

### 2.2. Study Population

Patients with a record of a STS pathology specimen in the period January 1, 2000 to December 31, 2007 were selected from the PALGA database. We excluded Kaposi's sarcomas (as these are strongly associated with herpesvirus [21], and the 2002 WHO classification of STSs does not classify these tumors as a true malignant sarcoma since there are major uncertainties as to their true biology [22]) and gastrointestinal stromal tumors (GIST; as these tumors are largely driven by activating mutations in the protooncogene *KIT*, with the protein tyrosine kinase inhibitor imatinib and sunitinib as specific treatments for this tumor [23]). Date of first STS pathology specimen was defined as the cohort entry date. To be able to define comorbidities in recent history, patients needed to have at least 12 months of history in the PHARMO RLS. All patients were followed from cohort entry until end of data-collection in the PHARMO RLS (i.e., the patient moves out of the PHARMO RLS catchment area), death, or end of the study period (December 31, 2008), whichever occurred first.

### 2.3. Cancer-Free Controls

The selection of the control cohort was stepwise. First, each STS patient was matched 1 : 20 by age and gender with a patient from the PHARMO RLS. Each of these potential controls was assigned the cohort entry date of the STS patient case match. Potential cases could not be selected as controls. Second, we eliminated from the pool of 20 possible controls, any control that did not have 12 months of history before cohort entry date in the PHARMO RLS and/or who had a hospitalization with a primary diagnosis for any cancer (ICD-9-CM codes: 140–239) in the period of ten years before the cohort entry date until December 31, 2008. Finally, out of the remaining controls that fulfilled the above criteria, ten were randomly selected and included in the control cohort.

### 2.4. Characteristics

We determined gender, age, and duration of followup of the patients included in our study cohort. Topography of the tumor was obtained from PALGA and classified as follows: extremities, head and neck, thorax, abdomen or (retro)peritoneum, and overlapping sites or sites not otherwise specified. Histologic type of STS was also obtained from PALGA and determined by using morphology ICD-O-3 codes (International Classification of Diseases for Oncology codes) [24–26]. In addition, data on treatment in the first six months after STS diagnosis was obtained from the PHARMO RLS. Surgery was assessed based on hospital procedure codes and the use of chemotherapy was defined based on hospital discharge diagnoses (ICD-9-CM code: V581) and/or oral chemotherapies supplied by community pharmacies (ATC code: L01). Data on radiation therapy was not available in the data sources of the PHARMO RLS.

### 2.5. Comorbidities

Comorbidities were defined via hospital admissions (ICD-9-CM codes) and/or drug dispensings (ATC codes) captured in the PHARMO RLS. Patients were considered to have a comorbidity if they either had been hospitalized for the condition or had been dispensed a medication used to treat the condition twice in a 365-day period. For the calculation of incidence rates, the event date of the first of each comorbidity was defined as the date of the hospitalization or the date of the first dispensing, whichever came first. The following comorbidities were considered: cardiovascular disease, including heart disease, thrombosis, and hypertension (ICD-9-CM code: 410, 411, 413, 414, 425–428, 433, 435 or ATC code: B01, C01–C04, C06–C10), respiratory disease, including asthma, and chronic obstructive pulmonary disease (ICD-9-CM code: 491–493, 496 or ATC code: R03), diabetes (ICD-9-CM code: 250 or ATC code: A10), anemia (ICD-9-CM code: 280, 281, 283–285 or ATC code: B03), or depression (ICD-9-CM code: 296.2, 296.3, 296.82, 298.0, 311, 300.4, 309.0, 309.1, 300.9, V62.84, 960–979, E950–E959, E980–E989 or ATC code: N06A). Comorbidities were assessed in 12 months, of history and during followup (0 to 6 months, 6 to 12 months and 12 months to total followup).

### 2.6. Analyses

Prevalence proportions in the 12 months before cohort entry were assessed for each comorbidity and expressed with accompanying 95% confidence intervals (CI). Conditional logistic regression was used to compare the prevalence of the comorbidities between the STS patients and the cancer-free control group. For incidence calculations, patients with a prevalent comorbidity (i.e., in the 12 months prior to cohort entry) were removed from the at-risk population for that specific event. Incidence rates per 1,000 person-years with accompanying 95% CI based on Byar's approximation [27] were calculated for the three distinct periods of followup time. Cox proportional hazards regression analysis was used to compare the incidence of the comorbidities between STS patients and their cancer-free controls. In addition, Cox proportional hazards regression models were fit to identify independent risk factors for developing cardiovascular disease, anemia, and depression (i.e., the three significantly increased comorbidities after STS diagnosis compared to cancer-free controls) in the complete followup after STS diagnosis. The patient characteristics mentioned under the subsection Characteristics were considered as potential risk factors. All risk factors associated with the outcome in the univariate analyses were included in the multivariate analyses. Statistical significance was defined at an alpha level of 0.05. Data were analyzed using SAS programs that are organized within SAS Enterprise Guide version 4.2 (SAS Institute Inc., Cary, NC, USA). Data management was conducted under UNIX using SAS version 9.2.

## 3. Results

A total of 533 patients with a STS resection specimen between 1 January 2000 and 31 December 2007 were selected from the PALGA database and linked to the PHARMO RLS (Table 1). Mean (± standard deviation: SD) age of these patients was 56 (±20) years and mean (±SD) followup in the PHARMO RLS was 3.1 (±2.3) years. Most STSs were situated in the extremities (36%), followed by the thorax (18%) and the abdomen or (retro)peritoneum (17%). For a large proportion of tumors, histology was not specified (*N* = 173, 32%). The most common known histological tumor type was leiomyosarcoma (23% (84/360)), followed by liposarcoma (21% (77/360)). Treatment in the first six months after STS diagnosis was mainly surgery (82%). Chemotherapy was administered to 10% of the patients included in our study cohort.

Table 2 presents the proportion of patients with comorbidity in the 12 months before STS diagnosis compared to cancer-free controls. The most prevalent comorbidity in STS patients was cardiovascular disease (33%), followed by respiratory disease (10%), diabetes (7%), anemia (6%), and depression (6%). Overall, STS patients were twice as likely as the cancer-free control group to have any of the comorbidities investigated. The highest odds ratio (OR) was found for anemia (OR = 3.6, 95% CI: 2.4–5.5).

Incidence rates of the comorbidities are presented for three distinct periods of followup time (Table 3). The incidence of cardiovascular disease, anemia and depression was highest during the first six months after STS diagnosis, 124, 85, and 40 per 1,000 person years (py), respectively, and decreased to 38, 11, and 13 per 1,000 py, respectively, in the period of 12 months to total followup. The incidence of respiratory disease and diabetes among STS patients remained stable during followup, ranging from 13–21 per 1,000 py for respiratory disease and 5–9 per 1,000 py for diabetes. Compared to the cancer-free control cohort, STS patients were at increased risk for cardiovascular disease (hazard ratio (HR) = 3.7, 95% CI: 2.1–6.3), anemia (HR = 6.9, 95% CI: 3.8–12.4), and depression (HR = 3.2, 95% CI: 1.5–6.9) during the first six months after diagnosis. This risk decreased over time, however, it was still significant for anemia (HR = 2.3, 95% CI: 1.2–4.4) and depression (HR = 2.0, 95% CI: 1.1–3.7) in the period of 12 months to total followup. Patients with STS had a similar risk of respiratory disease and diabetes compared to their cancer-free controls during the various followup periods, except for the period 12 months after diagnosis to total followup, where patients with STS had a two times increased risk of respiratory disease (HR = 2.2, 95% CI: 1.3–4.0).

Multivariate analyses were performed to determine the risk factors for developing cardiovascular disease, anemia and depression, that is, the three significantly increased comorbidities after STS diagnosis. Risk factors associated with the comorbidity in the univariate analyses are presented in Table 4 and were included in the multivariate analyses. Independent risk factors for cardiovascular disease after diagnosis for STS were male gender (HR = 1.9, 95% CI: 1.1–3.5) and STS located in abdomen or (retro)peritoneum (HR = 2.1, 95% CI: 1.1–4.6). The only independent risk factor for developing anemia after STS diagnosis was receiving chemotherapy during the first six months after STS diagnosis (HR = 1.9, 95% CI: 1.1–4.1).

## 4. Discussion

To our knowledge, this is the first population-based study to report on the prevalence of concomitant diseases at STS diagnosis and the incidence of comorbidities after STS diagnosis and treatment. We showed that 33% of the patients with STS suffered from cardiovascular disease (including hypertension) at time of diagnosis, and 10% had evidence of respiratory disease. Diabetes, anemia, and depression occurred in 6-7% of the patients at time of diagnosis. In a previously performed study [14] in The Netherlands on comorbid conditions among patients diagnosed with one of the 20 most frequent tumors, similar results were found. In that study cardiovascular disease (including hypertension) was present in 30% of the patients, respiratory disease in 10% of the patients and diabetes in 7% of the patients [14].

In the current study, STS patients were twice as likely to have any of the comorbidities prior to diagnosis compared with the cancer-free control group. This can likely be attributed to several risk factors that are both related to the development of the comorbidity and STS such as immunodeficiency, environmental factors, lifestyle factors, and genetic factors [8, 9].

Cardiovascular diseases, anemia, and depression were common incident comorbidities, especially in the first six months after STS diagnosis when rates were 124, 85, and 40 per 1,000 py, respectively, and 3–7 times higher than among cancer-free controls. These incidence rates decreased during followup, which suggests that cardiovascular diseases, anemia and depression are comorbidities related to the cancer itself or its treatment. This was investigated in our risk factor analysis. We found few significant independent risk factors, which might be explained by the relatively small patient cohort as STS is a rare tumor. We did find that male gender and STS located in the abdomen or (retro)peritoneum significantly increased the risk of developing cardiovascular disease after correction for other risk factors. A possible explanation for this finding is that abdominal or (retro)peritoneal STS tumors are mostly larger and of higher stage at presentation than those located elsewhere [10]. This may be due to the fact that these tumors arise in a clinically silent anatomical region where symptoms develop late in contrast to most head and neck tumors, which become clinically evident and symptomatic at a small size. Following this, STS tumors located in the abdomen might compress or invade other vital organs located in the abdomen. Second, treatment of abdominal STS tumors is more invasive and complicated. It is more difficult to successfully provide a complete operative resection, vital organs might be damaged due to the surgery or radiation therapy and following the more advanced stage at diagnosis, relatively more patients will receive chemotherapy [28].

In addition to risk factors for cardiovascular disease, a clear association was demonstrated between chemotherapy in the first six months after STS diagnosis and the risk of anemia during followup. It is well recognized that anemia might arise during or shortly after myelotoxic chemotherapy [29, 30]. Next to chemotherapy, radiation therapy may further negatively impact the severity and incidence of anemia [31]. Unfortunately we did not have data on radiotherapy available for all patients included in our study cohort. A former Dutch study on STS patients in the period 1989–1995 showed that 25% of the STS patients received radiotherapy with or without surgery and chemotherapy [24]. Analyses with data from a more recent cohort, the linked Eindhoven Cancer Registry-PHARMO cohort [32], showed that about 40% of the STS patients in the period 2000–2007 received radiotherapy.

Next to missing data on radiotherapy, another study limitation concerns the way the comorbidities were captured. In this study we did not use data from general practitioners or outpatient medical visits. The presence of comorbidities was based on hospitalizations and/or drug dispensings and consequently comorbid cases with a mild course were not included. Furthermore, identifying only the medically treated depression, limits the ability to directly compare these results with studies using structured clinical interviews or screening questionnaires to define depressive disorders. Regarding cardiovascular comorbidity, the definition used is very broad. However, low numbers of the patients included in this study did not allow sub classification of the results. Finally, as STSs are a heterogeneous group of tumors and there is a considerable variation in incidence patterns of sarcomas by histological type and anatomic site [25], future studies with larger patient numbers should focus on determining the comorbidities per histological subtype.

## 5. Conclusion

This study quantifies the significant burden of prevalent comorbidity in patients diagnosed with STS and presents the incidence of comorbidity after STS diagnosis and treatment. Prevalent comorbidity at STS diagnosis implies that more personalized supportive care is required for these patients. Incident comorbidity after diagnosis and treatment provides insight into the pharmacovigilance of STS treatment which can be used to improve the outcome of these patients.

## Figures and Tables

**Table 1:** Characteristics of newly diagnosed patients with soft tissue sarcoma.

Characteristics	Patients with soft tissue sarcoma (*N* = 533)
	*N* (%)
Gender	
Male	266 (50)
Female	267 (50)
Age in years	
<50	180 (34)
50–<70	211 (40)
≥70	142 (27)
mean ± SD	56 (±20)
Location	
Extremities	190 (36)
Head and neck	77 (14)
Thorax	97 (18)
Abdomen or (retro)peritoneum	91 (17)
Overlapping sites and NOS	78 (15)
Histology	
Fibrosarcoma (ICD-O-3: 8810-4)	17 (3)
Malignant fibrous hystiocytoma	28 (5)
(ICD-O-3: 8830)	
Dermatofibrosarcoma	27 (5)
(ICD-O-3: 8832-3)	
Liposarcoma (ICD-O-3: 8850-8)	77 (14)
Leiomyosarcoma (ICD-O-3: 8890-1)	84 (16)
Rhabdomyosarcoma	17 (3)
(ICD-O-3: 8900-1, 8910, 8920)	
Synovial sarcoma (ICD-O-3: 9040-3)	9 (2)
Hemangiosarcoma (ICD-O-3: 912,	22 (4)
913, 915)	
Malignant peripheral nerve sheath	16 (3)
tumor (ICD-O-3: 9540, 9560)	
Sarcoma, other	63 (12)
Sarcoma, NOS (ICD-O-3: 8800)	173 (32)
Treatment^1,2^	
Surgery	437 (82)
Chemotherapy	55 (10)

SD: standard deviation; NOS: not otherwise specified; ^1^no data on radiotherapy available; ^2^defined during the first six months after soft tissue sarcoma diagnosis.

**Table 2:** Proportion of patients with comorbidities* 12 months before diagnosis of soft tissue sarcoma, compared to a noncancer population.

Comorbidity	Patients with soft tissue sarcoma (*N* = 533)	Cancer-free control cohort (*N* = 5,330)	Odds ratio
	*N* (%)	*N* (%)	(95% CI)
Cardiovascular disease	178 (33)	1,148 (22)	2.0 (1.7–2.5)
Respiratory disease	54 (10)	262 (5)	2.2 (1.6–3.0)
Diabetes	36 (7)	212 (4)	1.8 (1.2–2.6)
Anemia	31 (6)	92 (2)	3.6 (2.4–5.5)
Depression	34 (6)	177 (3)	2.0 (1.4–2.9)

95% CI: 95% confidence interval; *comorbidity defined as either at least 2 dispensings of a relevant drug or a hospitalization for that condition before soft tissue sarcoma diagnosis.

**Table 3:** Incidence rates of comorbidities* stratified by followup time since diagnosis of soft tissue sarcoma, compared to a noncancer population.

	Patients with soft tissue sarcoma			Cancer-free control cohort			Hazard ratio
	Events	Followup	IR per 1,000	Events	Followup	IR per 1,000	(95% CI)
		PY	PY (95% CI)		PY	PY (95% CI)	
0 to 6 months after diagnosis							
Cardiovascular disease	20	161	124 (75–193)	72	2,004	36 (28–45)	3.7 (2.1–6.3)
Respiratory disease	3	220	14 (5–41)	30	2,444	12 (8–18)	1.1 (0.3–3.7)
Diabetes	2	226	9 (0–31)	9	2,473	4 (2–7)	2.5 (0.5–11.5)
Anemia	19	224	85 (49–134)	31	2,527	12 (8–17)	6.9 (3.8–12.4)
Depression	9	226	40 (18–75)	31	2,486	12 (8–18)	3.2 (1.5–6.9)
6 to 12 months after diagnosis							
Cardiovascular disease	8	143	56 (21–112)	52	1,841	28 (21–37)	2.6 (1.2–5.9)
Respiratory disease	4	195	21 (5–51)	16	2,265	7 (4–11)	2.5 (0.8–7.6)
Diabetes	1	202	5 (0–30)	13	2,298	6 (3–10)	0.8 (0.1–6.5)
Anemia	7	194	36 (15–72)	18	2,344	8 (5–12)	5.4 (2.1–13.6)
Depression	1	198	5 (0–30)	23	2,301	10 (7–15)	0.5 (0.1–3.8)
12 months after diagnosis to total followup							
Cardiovascular disease	29	762	38 (25–55)	292	10,594	28 (24–31)	1.4 (0.9–2.1)
Respiratory disease	14	1,042	13 (8–22)	78	13,491	6 (5–7)	2.2 (0.9–4.0)
Diabetes	9	1,106	8 (4–15)	69	13,804	5 (4–6)	1.6 (0.8–3.2)
Anemia	12	1,063	11 (6–20)	75	14,036	5 (4–7)	2.3 (1.2–4.4)
Depression	14	1,076	13 (7–21)	91	13,655	7 (5–8)	2.0 (1.1–3.7)

PY: person-years; IR: incidence rate; 95% CI: 95% confidence interval; *comorbidity defined as either at least 2 dispensings of a relevant drug or a hospitalization for that condition after soft tissue sarcoma diagnosis.

**Table 4:** Risk factors for developing cardiovascular disease, anemia, and depression after soft tissue sarcoma diagnosis.

Characteristics	Cardiovascular disease		Anemia		Depression	
	HR multivariate #	95% CI	HR multivariate #	95% CI	HR multivariate #	95% CI
Gender						
Male	1.9	(1.1–3.5)	1.4	(0.7–2.8)	1.0	(0.4–2.4)
Female	1	reference	1	reference	1	reference
Age in years						
<50	1	reference	1	reference	1	reference
50–<70	0.9	(0.4–1.8)	0.7	(0.3–1.8)	1.1	(0.6–2.4)
≥70	1.2	(0.5–2.5)	0.9	(0.3–2.4)	1.5	(0.7–3.5)
Location						
Extremities	1	reference				
Head and neck	0.8	(0.3–2.0)				
Thorax	1.4	(0.6–3.1)				
Abdomen or (retro)peritoneum	2.1	(1.1–4.6)				
Overlapping sites and NOS	1.9	(0.8–4.7)				
Treatment^1^						
Surgery	0.6	(0.2–2.3)	0.7	(0.2–2.6)		
Chemotherapy	1.7	(0.7–4.4)	1.9	(1.1–4.1)		

NOS: not otherwise specified; HR: hazard ratio; 95% CI: 95% confidence interval; # the multivariate model included all factors that were univariately associated with the outcome; ^1^defined during the first six months after soft tissue sarcoma diagnosis.
